# Identification of pyrin targets by CHiP-Seq

**DOI:** 10.1186/1546-0096-13-S1-P125

**Published:** 2015-09-28

**Authors:** G Wood, Y Kanno, H Sun, G Gutierrez-Cruz, I Aksentijevich, D Kastner

**Affiliations:** 1 NHGRI, MCIDB, Bethesda, USA; 2 NIAMS, LCBS/MIIB, Bethesda, USA; 3 NIAMS, Biodata Mining and Discovery Section/OST, Bethesda, USA; 4 NIAMS, Ultra High-Throughput DNA Sequencing/OST, Bethesda, USA

## Introduction

Familial Mediterranean fever (FMF) is a recessive disorder characterized by episodes of fever and neutrophil-mediated serosal inflammation. The gene causing FMF, *MEFV*, encodes a protein, pyrin. Pyrin is expressed predominantly in innate immune cells such as neutrophils, monocytes, and dendritic cells, but not in lymphocytes. Studies of pyrin localization show a cell-type dependency. Recent studies have demonstrated that the N-terminal fragment of cleaved pyrin binds to p65 and enhances its entrance into the nucleus. Also, we have previously shown by chromatin immunoprecipitation coupled with PCR (ChIP-qPCR) that in THP1 cells, pyrin can bind to the promoter of the transcription factor, IRF2.

## Objective

To further examine the hypothesis that pyrin binds to DNA and acts as a nuclear factor.

## Methods

THP1 cells were formaldehyde cross-linked and sonicated. Chromatin was immunoprecipitated with antibodies against pyrin or normal IgG as control. After immunoprecipitation, the DNA was purified and then sequenced on an Illumina HiSeq 2000. Sequencing reads were mapped to human genome hg18 and significant peaks were then called with the Model-based Alignment of ChIP-Seq (MACS) program. Peaks were assigned to genes if they are within promoters (TSS -/+ 5000 bases) or gene bodies. For ChIP-PCR assays, purified ChIP DNA samples were used for amplifications of specific regions of genomic DNA.

## Results

The initial ChIP-Seq was performed on two biological replicas. A total of 25,576,964 (IgG) and 22,124,860 (pyrin) nonredundant reads from replica-1 and 12,382,375 (IgG) and 10,612,526 (pyrin) nonredundant reads from replica-2 were aligned onto the human genome (hg18). We identified a total of 211 and 226 pyrin-occupied peaks, respectively. Peaks that hit in the same gene were further evaluated with the UCSC Genome Browser. Only one peak was shown to overlap in the two replicas. This peak corresponded to a gene involved in the nuclear pore complex, *NUP98.*With so few hits, another ChIP-Seq was done. We identified a total of 1343 peaks. We compared peaks from replica 1 and replica 2 with the new ChIP seq peaks to see if any of these peaks overlapped. This identified an additional 3 genes, *ATF6*, *IRF4,* and *TLR6*. Preliminary results by ChIP-PCR show enrichment of TLR6 after pyrin pull down.

## Conclusion

Our findings suggest that in THP1 cells pyrin binds to DNA and might act as nuclear factor. Further research is needed to validate the genes identified from ChIP-Seq and examine the downstream effect of pyrin-DNA binding.

